# Epistemic trust and personality functioning mediate the association between adverse childhood experiences and posttraumatic stress disorder and complex posttraumatic stress disorder in adulthood

**DOI:** 10.3389/fpsyt.2022.919191

**Published:** 2022-08-10

**Authors:** Hanna Kampling, Johannes Kruse, Astrid Lampe, Tobias Nolte, Nora Hettich, Elmar Brähler, Cedric Sachser, Jörg M. Fegert, Stephan Gingelmaier, Peter Fonagy, Lina Krakau, Sandra Zara, David Riedl

**Affiliations:** ^1^Department of Psychosomatic Medicine and Psychotherapy, Justus Liebig University Giessen, Giessen, Germany; ^2^Department for Psychosomatic Medicine and Psychotherapy, Medical Center of the Philipps University Marburg, Marburg, Germany; ^3^Ludwig Boltzmann Institute for Rehabilitation Research, Vienna, Austria; ^4^VAMED Rehabilitation Center, Schruns, Austria; ^5^Anna Freud National Centre for Children and Families, London, United Kingdom; ^6^Wellcome Trust Centre for Neuroimaging, Institute of Neurology, University College London, London, United Kingdom; ^7^Department of Psychosomatic Medicine and Psychotherapy, University Medical Center of the Johannes Gutenberg University Mainz, Mainz, Germany; ^8^Behavioral Medicine Research Unit, Integrated Research and Treatment Center for Adiposity Diseases, University Medical Center Leipzig, Leipzig, Germany; ^9^Department of Child and Adolescent Psychiatry/Psychotherapy, Ulm University, Ulm, Germany; ^10^Psychology and Diagnostics for Emotional and Social Development for the Emotionally Impaired, University of Education Ludwigsburg, Ludwigsburg, Germany; ^11^Department of Psychiatry, Psychotherapy, Psychosomatics and Medical Psychology, Medical University of Innsbruck, Innsbruck, Austria

**Keywords:** adverse childhood experiences, complex posttraumatic stress disorder, epistemic trust, mediator, personality functioning, posttraumatic stress disorder

## Abstract

**Background:**

Adverse childhood experiences (ACEs) are associated with posttraumatic and complex posttraumatic stress disorder symptoms in adulthood (PTSD/cPTSD), as well as reduced epistemic trust (trust in the authenticity and personal relevance of interpersonally transmitted information) and impaired personality functioning. The present work aims to investigate the predictive value of epistemic trust—the capacity for social learning—on the mediating effect of personality functioning in the association of ACEs and PTSD/cPTSD.

**Methods:**

We conducted structural equation modeling (SEM) based on representative data of the German population (*N* = 2,004). Personality functioning (OPD-SQS) was applied as a mediator between ACEs and PTSD/cPTSD (ITQ), while epistemic trust (ETMCQ) was added as predictor for OPD-SQS. TLI, CFI, and RMSEA (95%-CI) determined the models’ fit.

**Results:**

*N* = 477 (23.8%) participants reported at least one ACE and *n* = 218 (10.9%) reported ≥4 ACEs. Fit indices were good for both PTSD (TLI = 0.96; CFI = 0.99; RMSEA = 0.06; 95%CI: 0.041–0.078) and cPTSD (TLI = 0.96; CFI = 0.99; RMSEA = 0.06; 95%CI: 0.043–0.081). ACEs were significantly associated with cPTSD (β = 0.44, *p* < 0.001) and PTSD (β = 0.29, *p* < 0.001), explaining 20 and 8% of its variance. Adding personality functioning as a mediator increased the explained variance of cPTSD and PTSD to 47 and 19% while the direct association between ACEs and cPTSD/PTSD decreased (β = 0.21/β = 0.17), thus, indicating a partial mediation. Including epistemic trust substantially increased the explained variance for personality functioning (41%) compared to ACEs as a single predictor (16%).

**Conclusion:**

We add to previous research emphasizing the association between ACEs and PTSD/cPTSD symptoms. Offering insights on underlying mechanisms, we show that epistemic trust and personality functioning are relevant mediators. Since both are modifiable by psychotherapy, knowledge about the role of these constructs can inform research on psychotherapeutic interventions and prevention.

## Introduction

Adverse childhood experiences (ACEs) including child maltreatment and household dysfunction are a worldwide phenomenon (1), estimated to affect over 55 million children in Europe alone (2), and more specifically in Germany, about 44% (3) of the general population, making ACEs a global and highly prevalent problem. ACEs are defined as “childhood events, varying in severity and often chronic, occurring in a child’s family or social environment that cause harm or distress, thereby disrupting the child’s physical or psychological health and development” (4). There is a large body of evidence suggesting a close association between adverse experiences as a child and the development of physical health problems (5–8) and mental disorders (including psychological distress) (8–12) in adulthood.

With regard to mental disorders, posttraumatic stress disorder (PTSD) and borderline personality disorder (BPD) are most often associated with ACEs (13–17). However, specific symptoms often found in persons with PTSD and a history of ACEs led to significant conceptual changes. The ICD-11 considers different PTSD syndromes by adding a new diagnosis referred to as complex posttraumatic stress disorder (cPTSD), which is associated with repeated or prolonged experiences of traumatic events, resulting in specific additional symptoms such as impaired affect regulation, negative self-concept, and interpersonal problems (18, 19). The variety of PTSD symptoms (e.g., re-experiencing, avoidance, hyperarousal), additional specific cPTSD symptoms (e.g., impaired affect regulation), as well as frequent comorbidities (e.g., depressive symptoms or substance abuse) result in a very heterogeneous population of patients with PTSD and cPTSD, causing intense individual distress and are closely linked to serious disability as well as increased morbidity and mortality (20–24).

The close association between ACEs and the development of PTSD and cPTSD during adolescence or in adulthood emphasizes the importance of broadening our understanding of the underlying pathways through which ACEs and mental health issues are linked. Research focusing on ACEs and how they increase the risk for adult psychopathology proposed various potential mechanisms including brain structure and function, epigenetic processes, gene expression to neuroendocrine, immune and neurotransmitter systems, as well as social cognition (25–27). Another focus has been on personality development which has gained much interest in recent empirical research. Patients with a history of ACEs often show e.g., impaired identity perception, interpersonal difficulties, or altered affect regulation (28). Given that experiences made in early childhood are essential for the development of personality functioning by shaping basic mental capacities or adaptive coping behaviors (29–32), personality functioning might be a pathway linking ACEs and adult psychopathology. Personality functioning (also referred to as “structure”) describes a person’s abilities directed toward the self (identity perception, self-regulation) and others (empathy, intimacy) in four domains related to capacities of cognition/perception, regulation, communication, and attachment (33). Since personality disorders are closely linked to impaired personality functioning, the concept of personality functioning has been included as a dimensional measure of basic psychological capacities in both the DSM-5 (34) as well as the ICD-11 (35–37). The ICD-11 represents a radical change in the classification of personality disorders where former categorical descriptions are now replaced by a dimensional structure. Diagnoses of personality disorders are conducted in two stages by assessing (1) the severity level and (2) domain traits. Here, special regard has been given to the borderline concept that can be optionally assigned as a “borderline pattern” after severity levels have been determined (35). With regard to the also newly introduced diagnosis cPTSD, this becomes particularly interesting as both diagnoses show substantial overlaps, specifically in terms of self- and interpersonal problems (38). The ICD-11 allows for this by making it possible to assign both diagnoses at the same time. While both are strongly associated with ACEs, the diagnosis of cPTSD actually requires the presence of trauma and PTSD symptoms. Both diagnoses are to be distinguished by specific elements. While cPTSD “typically involves stable and persistent patterns of negative self-perception while emphasizing avoidant interpersonal patterns,” the borderline pattern differs by allowing “an unstable or internally contradictory sense of self, which may involve both overly negative and overly positive self-views” (38).

Secure relationships and attachments alongside adequate emotional mirroring processes facilitate the development of personality functioning in early childhood (39). Given that personality functioning develops at a young age (29, 40, 41), it becomes obvious that ACEs (e.g., in form of maltreatment carried out by care-givers or attachment disruptions) might hamper and disrupt this developmental processes (42, 43), and thus, result in impaired personality functioning. Based on pioneering work by Sperber et al. (44) and Wilson and Sperber (45), Fonagy and Allison emphasize that secure attachment relationships are not only highly relevant for the development of personality functioning in infancy, but also for the development of the capacity of epistemic trust (46–48). Epistemic trust describes the “trust in the authenticity and personal relevance of interpersonally transmitted knowledge about how the social environment works and how best to navigate it” (49), or in other words, an “individual’s willingness to consider new knowledge from another person as trustworthy, generalizable, and relevant to the self,” in short: for social learning (47). In case of ACEs—especially if they occur in severe and regular form—a child’s early environment is characterized by unreliable or even malevolent caregiving experiences causing disrupted learning about the social world, and thus, resulting in a breakdown or underdevelopment of epistemic trust. Epistemic mistrust can cause uncertainty and continued epistemic vigilance that can manifest as the overinterpretation of other people’s motives (48, 49). In a state of epistemic hypervigilance, one will assume that the other’s intentions are deviating from those declared, having them treat the source of the information as not deferential (48). Also, the content of the information may be rejected and its meaning confused or misinterpreted as being malignant. Such failings in social communication might originate from epistemic hypervigilance, epistemic mistrust, or even epistemic freezing, with the latter describing the inability to trust others as a source of information regarding the world and its workings (49). In short, “epistemic mistrust manifests as the misattribution of intention and the assumption of malevolent motives behind another person’s actions, and therefore treating them with epistemic hypervigilance (or conversely, in some instances, excessive inappropriate epistemic trust or credulity)” (48). Fonagy and colleagues suggest that these might be characteristics of many patients with trauma and personality problems, and that many “types of psychopathology might be characterized by temporary or permanent disruption of epistemic trust and the social learning process it enables” (49).

Following this line of thinking, epistemic trust might have a relevant predictive influence on a person’s level of personality functioning. However, to our knowledge there is no research assessing the role of epistemic trust in the association between ACEs, personality functioning and PTSD and cPTSD respectively. Therefore, the present work aims to investigate the predictive value of reduced epistemic trust on the mediating effect of impaired personality functioning in the association between ACEs and PTSD and cPTSD. We hypothesize, (I) that in line with previous research, experiences of ACEs relate to higher rates of PTSD and cPTSD in the general population, (II) that ACEs correspond to greater impairment in personality functioning and epistemic trust, and finally (III) that personality functioning is a mediator regarding the association between ACEs and PTSD as well as cPTSD, and that epistemic trust will be a relevant predictor for personality functioning.

## Materials and methods

### Sample and setting

The present study is based on data from a representative sample of the German population collected by the independent demography research institute USUMA Berlin. Face-to-face interviews and self-report questionnaires were administered by trained interviewers between December 2020 and March 2021, yielding a total of *N* = 2,519 participants. Households within 258 predefined regions were selected by a random route procedure. In households with multiple persons, one person was randomly selected using the Kish-Selection-Grid. Inclusion criteria were sufficient German language skills, an age ≥ 16 and informed consent before taking part in the study (in the case of minors, informed consent was also obtained from a parent/legal guardian). The survey was conducted in accordance with the Declaration of Helsinki and fulfilled the ethical guidelines of the International Code of Marketing and Social Research Practice of the International Chamber of Commerce and the European Society of Opinion and Marketing Research. Regarding the beginning Covid-19-pandemic in Germany, all applicable hygiene regulations at that time were followed. Ethical approval was obtained by the Ethics Committee of the Medical Faculty of the University of Leipzig (no. 474/20-ek).

### Measures

#### Adverse childhood experiences questionnaire

ACEs were assessed using the ACEs Questionnaire (50), which is a widely used self-report tool for retrospectively evaluating numerous early childhood adversities. It comprises 10 items regarding abuse (emotional, physical, and sexual), neglect (emotional and physical), separation of a parent, violence against the mother, as well as problems of a household member (substance use, mental disorder, and prison stay). Each item is answered with either yes (1) or no (0), resulting in a sum score between 0 and 10. The German version of the ACE has shown acceptable reliability, with Cronbach’s α = 0.76 (51). In our sample, a good internal consistency of the ACE items could be observed (α = 0.81).

#### International trauma questionnaire

The ITQ is a brief self-report questionnaire to measure PTSD and cPTSD symptoms after a stressful life experience, also assessing whether diagnostic criteria of PTSD and cPTSD diagnoses according to ICD-11 are fulfilled (52). It comprises 18 items with response options ranging from 0 = “not at all” to 4 = “extremely.” The three PTSD core symptom clusters (re-experiencing, avoidance, and sense of threat) and the additional three areas related to disturbances in self-organization (DSO) (affective dysregulation, negative self-concept, and problematic relationships) are measured by two items each. For a dimensional assessment, the three items for the PTSD core symptom clusters and DSO, respectively, can be summed up resulting in an ITQ-PTSD sum score and an ITQ-cPTSD sum score, respectively, ranging from 0 to 24. To assess a probable PTSD diagnosis, functional impairment for the PTSD core symptoms are considered with additional three items. A probable PTSD diagnosis is met if at least one item in each core symptom cluster and one item of functional impairment for PTSD is answered with ≥2 (“moderately”). A probable cPTSD diagnosis is met, if PTSD criteria are satisfied and additionally at least one item in each DSO area is answered with ≥2 as well as and one item of functional impairment for DSO is answered with ≥2. According to ICD-11 diagnostic guidelines, a person may receive a possible diagnosis for PTSD or cPTSD, but not both. For both scales, reliability was good with α = 0.84 for the PTSD scale and α = 0.88 for the cPTSD scale (53). The German version has been validated in a representative population-based sample and can be used for research and clinical practice (54). In our sample, good internal consistency was observed for both the PTSD (α = 0.89) and cPTSD scale (α = 0.87).

#### Operationalized psychodynamic diagnosis structure questionnaire-short form

The Operationalized Psychodynamic Diagnosis Structure Questionnaire-Short Form (OPD-SQS) (55) is a 12-item self-report questionnaire to assess the level of personality functioning. A total score ranging from 0 to 48 and a score for each of the three subscales (self-perception, interpersonal contact, and relationship model) ranging from 0 to 12 can be calculated. Higher values indicate more severe deficits in personality functioning. Good validity and reliability have been reported for the total scale, with Cronbach’s α = 0.88 (55). In our sample, good internal consistency was observed for the OPD-SQS total score (α = 0.91).

#### Epistemic trust, mistrust and credulity questionnaire

The German Version of the Epistemic Trust, Mistrust and Credulity Questionnaire (ETMCQ) (56, 57) was used to assess the participants’ levels of trust in communicated knowledge, i.e., epistemic trust. The ETMCQ consists of 15 items to measure the three independent subscales of the epistemic trust construct: epistemic trust, mistrust, and credulity. Examples for the respective scales are “I find information easier to trust and absorb when it comes from someone who knows me well” for epistemic trust, “If you put too much faith in what people tell you, you are likely to get hurt” for epistemic mistrust, and “When I speak to different people, I find myself easily persuaded even if it is not what I believed before” for epistemic credulity. Each item has a response option ranging from 1 = “strongly disagree” to 7 = “strongly agree,” resulting in a sum score from 15 to 105. High trust reflects a persons’ ability to be open to opportunities for social learning in relationships, while high mistrust indicates a tendency to treat information sources as unreliable and to rather avoid being influenced by communication from others. High credulity reflects a persons’ lack of clarity about its own position, which can lead to high vulnerability to misinformation and exploitation by others. For the ETMCQ, good reliability and validity have been reported, with the internal consistency for the full scale ranging from Cronbach’s α = 0.71 to α = 0.78. In our sample, good internal consistency was observed for the ETMCQ trust (α = 0.81) and credulity (α = 0.80) subscale, while the values for the mistrust subscale were somewhat questionable (α = 0.69).

### Statistical analyses

Demographics for the sample are presented with means and standard deviations (SD). Patients with >50% missing items in the ITQ, ACE scale, OPD-SQS or ETMCQ were excluded from the analysis. Sociodemographic and clinical data of the excluded sample was compared to the study sample using independent sample *t*-tests and χ^2^-tests. The effect of group differences was estimated using Hedges g’ for metric and ϕ for nominal data. Values of g’ = 0.2/ϕ = 0.1, g’ = 0.5/ϕ = 0.3 and g’ = 0.8/ϕ = 0.5 represent small, medium and large effect sizes, respectively.

**FIGURE 1:**
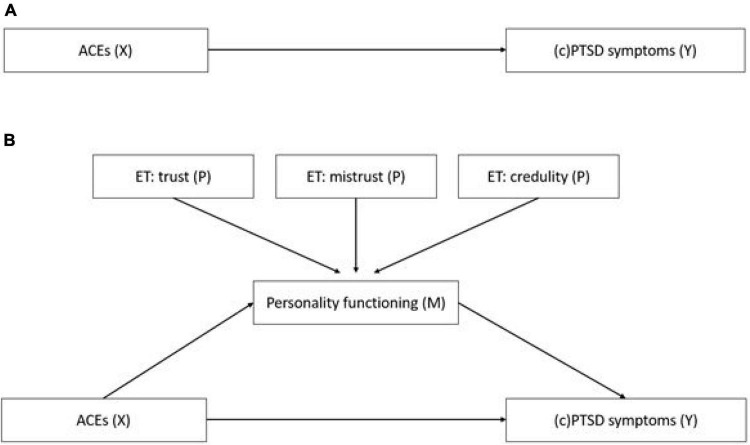
Structural equation models to test the mediation effect of personality functioning and epistemic trust on the relationship of ACEs with PTSD/cPTSD symptoms in adulthood. **(A)** Direct association of ACEs (X) and PTSD symptoms (Y). **(B)** Association of ACEs (X) and PTSD/cPTSD symptoms (Y), mediated by personality functioning (M), which was predicted by epistemic trust (P). Model A depicts the direct association of the independent variable ACEs (X) with the dependent variables PTSD and cPTSD respectively (Y). Model B depicts the model with personality functioning as a mediator (M) and the three epistemic trust subscales as predictors (P) for the mediator variable.

The relationships between ACEs, epistemic trust, personality functioning, and PTSD/cPTSD symptoms were investigated with structural equation models (SEM; see Figure 1). Missing data (<50% missing items) was imputed using the full information maximum likelihood (FIML) estimation, which is the default approach in AMOS. In model A, the direct influence of ACEs on PTSD/cPTSD symptoms in adulthood was tested. In model B, personality functioning as measured by the OPD-SQS total score was added to the model as a mediator for this relationship and the epistemic trust subscales were added as predictors for personality functioning. For sensitivity analyses, the model was also tested (a) in the complete sample with the assumption that all missing values equal the lowest possible symptom score and (b) in all participants without missing data in the ITQ cPTSD scale with the assumption that missing values in the ITQ PTSD scale represent the lowest possible symptom score.

To account for non-normal distribution of data, bootstrapped confidence intervals [5,000 samples, 95% confidence interval (CI)] were calculated to evaluate the statistical significance of all included paths in the SEM. To determine the model’s goodness of fit, Pearson’s chi-squared test (χ^2^), the comparative fit index (CFI), Tucker-Lewis Index (TLI), and root mean square error of approximation (RMSEA) with lower and higher bounds of the 95%-CI were calculated. To evaluate whether the empirical data was closely fitting the theoretical model, the *p*-value of Close Fit (PCLOSE) was calculated based on the RMSEA values, with values of *p* > 0.05 indicating close fit and *p*< 0.05 indicating worse than close model fit. Acceptable goodness of fit was defined as RMSEA values of <0.08 and CFI/TLI values >0.90. *p*-values < 0.05 (two-sided) were considered statistically significant. Statistical analyses were performed with IBM SPSS (v22.0) and SPSS AMOS (v24.0).

## Results

A total of 2,519 persons participated in the study. Of these, *n* = 515 participants (20.4%) were excluded because of missing data in the ITQ (mainly the PTSD symptom scale), the ACE questionnaire, the OPD-SQS, or the ETMCQ. The remaining *n* = 2,004 patients were included in the final analysis. Participants’ mean age was 51.3 years. The majority was female (52.5%), married (45.5%), had an education ranging from 10 to 13 years of school (57.1%), and were employed full time (41.4%). Most participants earned a net monthly household income between 1,500 and 2,499 € (31.7%). For more details on sociodemographic characteristics see Table 1.

**TABLE 1:** Sociodemographic characteristics—*N* = 2,004.

	*N*	(%)
**Sex**		
Male	947	(47.3)
Female	1,053	(52.5)
Diverse	4	(0.2)
**Age (*M* = 51.3; *SD* = 18.1)**		
<30	325	(16.2)
30–39	245	(12.2)
40–49	307	(15.3)
50–59	418	(20.9)
60–69	348	(17.4)
>70	361	(18.0)
**Education**		
<10 years	605	(30.2)
10–13 years	1,145	(57.1)
>13 years	202	(10.1)
Other qualification	35	(1.7)
Missing	17	(0.8)
**Relationship**		
Married	911	(45.5)
Single	574	(28.6)
Divorced	291	(14.5)
Widowed	221	(11.0)
Missing	7	(0.3)
**Employment status**		
Full time	830	(41.4)
Part time	250	(12.5)
Unemployed	161	(8.0)
In training	112	(5.6)
Retired	626	(31.2)
Missing	25	(1.2)
**Monthly net household income**		
<1,500 €	471	(23.5)
1,500–2,499 €	636	(31.7)
2,500–3,499 €	457	(22.8)
>3,500 €	440	(22.0)

Excluded patients were significantly younger (46.3 vs. 51.3 years; *p* < 0.001; g’ = 0.28), more often full time employed (51.0 vs. 41.6%) and less often retired (19.3 vs. 31.4%) (χ^2^ = 33.35, *p* < 0.001; ϕ = 0.12), more often single (35.6 vs. 28.7%) and less often widowed (4.3 vs. 11.1%) (χ^2^ = 26.08, *p* < 0.001; ϕ = 0.10), had been in school longer (χ^2^ = 11.66, *p* = 0.009; ϕ = 0.07), and reported less ACEs (0.6 vs. 1.0; *p* < 0.001; g’ = 0.24). However, all differences were of small effect size. No significant difference was observed for gender (χ^2^ = 1.06, *p* = 0.59; ϕ = 0.20) and monthly net household income (χ^2^ = 19.14, *p* = 0.12; ϕ = 0.09).

In addition, Table 2 shows the mean number of ACEs experienced by the participants of the included sample as well mean values regarding personality functioning, epistemic trust, and PTSD and cPTSD symptoms.

**TABLE 2:** Mean numbers of ACEs as well as mean values of ITQ (symptoms of PTSD and cPTSD, respectively), OPD-SQS (personality functioning), and ETMCQ (epistemic trust)—*N* = 2,004.

	*M*	(*SD*)
ACE	1.0	(1.8)
ITQ		
PTSD	2.8	(4.2)
cPTSD	4.2	(5.7)
OPD-SQS		
total score	24.1	(9.4)
self-perception	6.3	(3.1)
interpersonal contact	7.9	(3.4)
relationship model	10.0	(4.3)
ETMCQ		
trust	24.7	(5.4)
mistrust	14.5	(4.5)
credulity	12.5	(5.1)

ACE, Adverse Childhood Experiences Questionnaire; ITQ, International Trauma Questionnaire; PTSD, posttraumatic stress disorder; cPTSD, complex posttraumatic stress disorder; OPD-SQS, Operationalized Psychodynamic Diagnosis Structure Questionnaire-Short Form; ETMCQ, Epistemic Trust, Mistrust and Credulity Questionnaire.

### Prevalence and association of adverse childhood experiences and posttraumatic stress disorder as well as complex posttraumatic stress disorder in adulthood

A total of 1,309 (65.3%) participants reported no ACEs, while 477 (23.8%) had experienced 1–3 ACEs and the remaining 218 (10.9%) four or more ACEs [i.e., polytraumatized persons (58)]. The rates for PTSD and cPTSD symptoms above the cut-off were quite similar with a prevalence of 1.4% (*n* = 29) and 2.5% (*n* = 51) respectively.

A higher ACE score was significantly associated with higher scores for symptoms of PTSD (*r* = 0.31, *p* < 0.001) and cPTSD (*r* = 0.44, *p* < 0.001). Patients who were polytraumatized (i.e., four or more ACEs) in their childhood had 7.0-times increased likelihood to develop clinically relevant PTSD-symptoms (95%-CI: 4.0–12.1; *p* < 0.001) and a 14.8-times increased risk for clinically relevant cPTSD symptoms (95%-CI: 8.2–26.6; *p* < 0.001).

### The association between personality functioning and epistemic trust with adverse childhood experiences and posttraumatic stress disorder as well as complex posttraumatic stress disorder in adulthood

In our sample, ACEs were significantly associated with lower personality functioning as well as higher scores for epistemic mistrust and epistemic credulity as well as lower scores for epistemic trust. Additionally, participants with lower personality functioning reported higher PTSD and cPTSD scores. As for the epistemic trust subscales, higher epistemic credulity and mistrust were both significantly associated with higher PTSD and cPTSD symptoms. However, while lower epistemic trust was also associated with higher cPTSD symptoms, there was no significant association with PTSD symptoms (see also Table 3).

**TABLE 3:** Correlations between the ETMCQ, ACE, OPD-SQS, ITQ-PTSD, and ITQ-cPTSD scales.

	ACE	ITQ-PTSD	ITQ-cPTSD
ETMCQ: trust	–0.10***	–0.03	–0.16***
ETMCQ: mistrust	0.19***	0.14***	0.32***
ETMCQ: credulity	0.25***	0.24***	0.39***
OPD-SQS: total score	0.41**	0.40**	0.66**
OPD-SQS: self-perception	0.37***	0.45***	0.67***
OPD-SQS: interpersonal contact	0.37***	0.34***	0.61***
OPD-SQS: relationship model	0.34***	0.29***	0.49***

**p < 0.01; ***p < 0.001. ETMCQ, Epistemic Trust, Mistrust and Credulity Questionnaire; OPD-SQS, Operationalized Psychodynamic Diagnosis Structure Questionnaire-Short Form; ACEs, Adverse Childhood Experiences Questionnaire; ITQ, International Trauma Questionnaire; PTSD, posttraumatic stress disorder; cPTSD, complex posttraumatic stress disorder.

### Personality functioning and epistemic trust as mediators of the relationship of adverse childhood experiences with posttraumatic stress disorder and complex posttraumatic stress disorder symptoms in adulthood

In the first step, the direct associations of ACEs with PTSD and cPTSD symptoms in adulthood were investigated by calculation of a SEM. ACEs significantly predicted PTSD (*p*< 0.001, β = 0.31, 95%-CI: 0.26–0.36) and cPTSD (*p*< 0.001, β = 0.44, 95%-CI: 0.39–0.49) symptoms and explained 8 and 20% of the variance, respectively. Since the number of distinct sample moments was equal to the number of distinct parameters to be estimated (i.e., resulting in zero degrees of freedom), no model fit indices could be calculated.

**FIGURE 2:**
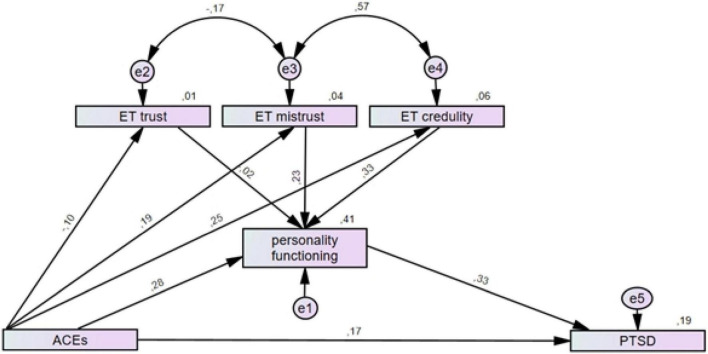
Structural equation models for the mediation effect of personality functioning and epistemic trust on the relationship of ACEs with PTSD symptoms. Personality functioning was added as a mediator for the association between ACEs and PTSD and the three epistemic trust subscales as predictors of personality functioning. Rectangles represent variables (ACEs, Adverse Childhood Experiences measured by the ACE; personality functioning measured by the OPD-SQS; ET, epistemic trust measured by the ETMCQ; PTSD, posttraumatic stress disorder symptoms measured by the ITQ) and circles represent error terms (e). Numbers next to arrows in the model represent standardized estimates, numbers next to factors represent the R2, i.e. the explained variance.

**FIGURE 3:**
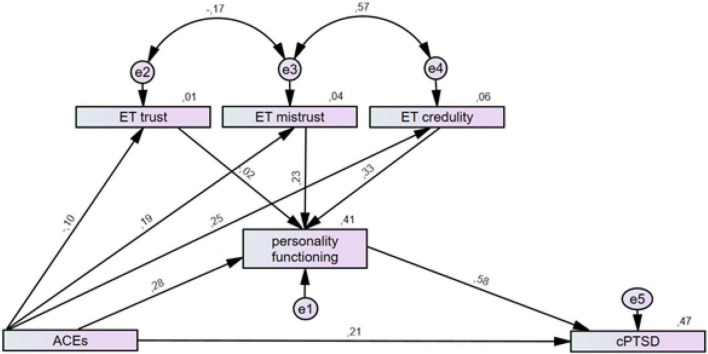
Structural equation models for the mediation effect of personality functioning and epistemic trust on the relationship of ACEs with cPTSD symptoms. Personality functioning was added as mediator for the association between ACEs and cPTSD and the three epistemic trust subscales as predictors of personality functioning. Rectangles represent variables (ACEs, Adverse Childhood Experiences measured by the ACE; personality functioning measured by the OPD-SQS; ET, epistemic trust measured by the ETMCQ; cPTSD, complex posttraumatic stress disorder symptoms measured by the ITQ) and circles represent error terms (e). Numbers next to arrows in the model represent standardized estimates, numbers next to factors represent the R2, i.e. the explained variance.

In the second step, the OPD-SQS total score was added as a mediator of the relationship between ACEs and PTSD/cPTSD symptoms, respectively, and the ETMCQ subscales were added as predictors for personality functioning. The overall explained variance substantially increased for PTSD (19%) and cPTSD (47%) and the direct association of ACEs with both PTSD (β = 0.17, 95%-CI: 0.12–0.23; *p* < 0.001) and cPTSD symptoms (β = 0.21, 95%-CI: 0.16–0.26; *p* < 0.001) was weakened. Epistemic mistrust (β = 0.23, 95%-CI: 0.18–0.28; *p*< 0.001) and epistemic credulity (β = 0.33, 95%-CI: 0.29–0.38; *p*< 0.001) significantly predicted the OPD-SQS total score and explained 41% of the variance (for details, see Figures 2, 3).

A good model fit was found for the model with PTSD (χ^2^ = 31.56, *p*< 0.001; CMIN/DF = 7.89; CFI = 0.99; TLI = 0.96; RMSEA = 0.059, 95%-CI: 0.041–0.078; PCLOSE = 0.20) and cPTSD as dependent variables (χ^2^ = 34.13, *p*< 0.001; CMIN/DF = 8.53; CFI = 0.99; TLI = 0.96; RMSEA = 0.061, 95%-CI: 0.043–0.081; PCLOSE = 0.14).

For sensitivity analyses, both models were also calculated with (a) all missing items imputed as lowest possible score on the ITQ scales and (b) missing ITQ-PTSD items imputed as lowest possible scores for all participants with complete ITQ-cPTSD items. Since these calculations showed no difference in results, the initial analyses were considered reliable.

## Discussion

Based on data of a representative survey of the German population, we examined the role of epistemic trust in the association between ACEs, personality functioning and PTSD as well as cPTSD, respectively.

Our results show that a third of our sample suffer from ACEs (about 24% had one to three and about 11% four or more ACEs), and about 2.5% and 1.4% fulfilled self-reported PTSD and cPTSD criteria respectively. Higher ACE scores were significantly associated with higher scores for symptoms of PTSD and even more so for symptoms of cPTSD. In addition, multiple experiences of ACEs (>4 ACEs) were associated with a severely increased risk to develop PTSD (7.0-times) and cPTSD (14.8-times) in adulthood. We add to previous research demonstrating an association of ACEs and PTSD as well as cPTSD, emphasizing the importance of a persons’ history of child maltreatment and adverse experiences for developing these psychopathologies (13, 15, 17, 59).

Consistent with our second hypothesis, we show that ACEs do indeed correspond to impairments in personality functioning but also higher epistemic mistrust and epistemic credulity, as well as reduced epistemic trust. Moreover, we demonstrate that impaired personality functioning as well as higher epistemic mistrust and epistemic credulity were significantly associated with increased PTSD and cPTSD scores, implying the possible importance of these constructs for the development of such psychopathology. With regard to personality functioning, this is in line with previous research demonstrating associations between personality functioning and PTSD and cPTSD that were also more pronounced for cPTSD than PTSD (60).

Following our initial line of thinking that the underlying pathway of the association between ACEs and PTSD as well as cPTSD might involve constructs such as personality functioning or epistemic trust, we included both variables in a SEM in order to explore the mediating and predictive characteristics of these constructs. For PTSD, the explained variance increased notably from 8 to 19% when including personality functioning as a mediator in the direct association between ACEs and PTSD. For cPTSD, the relationship was even stronger with the explained variance increasing from 20 to 47%. To our knowledge, this is the first study to investigate this association. Whilst previous research has focused on isolated and specific mechanisms such as affect regulation (28) or attachment (61), we assessed different but intertwined psychological functions comprising self-regulation, self-perception, and interpersonal difficulties in the form of personality functioning. Scarce research already including personality functioning focused on different psychopathologies in adulthood. For example, a similar pathway was found for depression and anxiety where personality functioning (partially) mediated the association between child maltreatment and depression and anxiety symptoms, respectively (12, 25). Personality functioning appears to be a relevant underlying mechanism in adults with different psychopathologies including PTSD and especially cPTSD and a history of ACEs.

We also included epistemic trust in our SEM to empirically test the predictive value of this construct. In line with our third hypothesis, our results show that, compared to ACEs as a single predictor, the inclusion of epistemic trust substantially increased the explained variance of personality functioning from 16 to 41%. These results suggest that epistemic trust or rather disruptions of epistemic trust have an important influence on personality functioning, and thus, might play a role in better understanding the implications of ACEs in those with PTSD and cPTSD. There is the possibility that the disruption of the system of trusting socially conveyed information might lead to problems of personality functioning because updating knowledge about the self becomes a challenge. In other words, the risk of PTSD and cPTSD increases because the person is less connected to their social network so that adequate personality functioning—which assumes a free-flow of information within a social network—becomes compromised. Yet, notably stronger associations for cPTSD compared to PTSD have to be considered with respect to the different diagnostic requirements: while cPTSD is normally associated with prolonged or multiple ACEs, PTSD can be caused by a variety of single stressful or threatening events. That ACEs might cause disruptions in epistemic trust and therefore impairments in personality functioning—an assumption that requires further research—might in part explain the specific symptoms accompanying cPTSD, namely impaired affect regulation, low self-esteem, and interpersonal problems (18, 19, 62).

Even though future research has to validate our findings, we believe knowledge about constructs such as personality functioning and specifically epistemic trust—both modifiable by psychotherapy (47, 62–64)—should inspire further research on psychotherapeutic prevention and intervention when addressing PTSD and more importantly cPTSD but also when considering adult psychopathology in general and personality disorders in particular.

## Strengths and limitations

A major strength of this study was the availability of representative data from a face-to-face survey which included information about participants’ psychological, physical, personal and socio-demographic characteristics. In addition, to our knowledge this is the first study to empirically examine the role of epistemic trust in the direct context of ACEs and PTSD as well as cPTSD in adulthood. While standardized clinical diagnoses are certainly the gold standard, we consider it a strength of the study that the layout of the ITQ allows for potential classification of diagnosis by assessing the specific diagnostic criteria of PTSD and cPTSD according to ICD-11 (52). Nevertheless, the ITQ is a self-report measure that does not resemble the quality of a clinical diagnosis. Moreover, there are potential limitations that should be considered with regard to the study results. While overall quality of the data is high (unbiased general population based data), the cross-sectional study design limits the interpretation of the results in terms auf causality. From a developmental perspective, ACEs could have also immediately resulted in disrupted trust and attachments, and therefore, PTSD or cPTSD in childhood, which in turn could have impaired a healthy personality development during late childhood and adolescence. While we examined epistemic trust in relation to personality functioning, we did not include attachment which might offer even further insights on the involved pathways of ACEs and adult psychopathology (43). In addition, we did not conduct separate analyses for different subtypes of ACEs that might yield further insights on the role of a specific type of ACEs—e.g., sexual abuse—in relation to adult psychopathology.

## Conclusion

Our results add to the body of evidence demonstrating the mediating effect of personality functioning in the association of ACEs and adult psychopathology. Our findings imply that personality functioning might play an important role in developing PTSD and even more importantly cPTSD symptoms in adulthood following ACEs. Theory driven and based on preliminary research in the area of personality research, we included epistemic trust as a new and potentially relevant element for this association, showing that epistemic trust had indeed a predictive influence on a persons’ level of personality functioning. This knowledge helps us to better understand the underlying pathways resulting in psychopathology following ACEs but also might inform psychotherapeutic treatment planning by considering and addressing the interfering role of e.g., epistemic mistrust within the therapeutic relationship.

## Data availability statement

The raw data supporting the conclusions of this article will be made available by the authors, without undue reservation.

## Ethics statement

The studies involving human participants were reviewed and approved by the Ethics Committee of the Medical Faculty of the University of Leipzig (474/20-ek). Written informed consent to participate in this study was provided by the participants’ legal guardian/next of kin.

## Author contributions

HK, DR, JK, AL, and TN participated in the research design. PF offered the conceptual framework of the epistemic trust construct. EB collected the data. DR conducted the formal statistical analyses. JK and AL supervised the study. HK and DR wrote the original draft of the manuscript. NH, LK, TN, and SZ contributed to the writing of the manuscript. NH, EB, CS, JK, AL, SG, PF, TN, LK, and SZ reviewed and edited the manuscript. All authors approved the final version of the manuscript.
