# Corrigendum: Epistemic trust and personality functioning mediate the association between adverse childhood experiences and posttraumatic stress disorder and complex posttraumatic stress disorder in adulthood

**DOI:** 10.3389/fpsyt.2024.1427500

**Published:** 2024-05-21

**Authors:** Hanna Kampling, Johannes Kruse, Astrid Lampe, Tobias Nolte, Nora Hettich, Elmar Brähler, Cedric Sachser, Jörg M. Fegert, Stephan Gingelmaier, Peter Fonagy, Lina Krakau, Sandra Zara, David Riedl

**Affiliations:** ^1^ Department of Psychosomatic Medicine and Psychotherapy, Justus Liebig University Giessen, Giessen, Germany; ^2^ Department for Psychosomatic Medicine and Psychotherapy, Medical Center of the Philipps University Marburg, Marburg, Germany; ^3^ Ludwig Boltzmann Institute for Rehabilitation Research, Vienna, Austria; ^4^ VAMED Rehabilitation Center, Schruns, Austria; ^5^ Anna Freud National Centre for Children and Families, London, United Kingdom; ^6^ Wellcome Trust Centre for Neuroimaging, Institute of Neurology, University College London, London, United Kingdom; ^7^ Department of Psychosomatic Medicine and Psychotherapy, University Medical Center of the Johannes Gutenberg University Mainz, Mainz, Germany; ^8^ Behavioral Medicine Research Unit, Integrated Research and Treatment Center for Adiposity Diseases, University Medical Center Leipzig, Leipzig, Germany; ^9^ Department of Child and Adolescent Psychiatry/Psychotherapy, Ulm University, Ulm, Germany; ^10^ Psychology and Diagnostics for Emotional and Social Development for the Emotionally Impaired, University of Education Ludwigsburg, Ludwigsburg, Germany; ^11^ Department of Psychiatry, Psychotherapy, Psychosomatics and Medical Psychology, Medical University of Innsbruck, Innsbruck, Austria

**Keywords:** adverse childhood experiences, complex posttraumatic stress disorder, epistemic trust, mediator, personality functioning, posttraumatic stress disorder

In the published article, there was an error. The prevalence rates for PTSD and CPTSD were not correctly printed.

A correction has been made to the **Results** section, *Prevalence and association of adverse childhood experiences and posttraumatic stress disorder as well as complex posttraumatic stress disorder in adulthood*, Paragraph 1.

This sentence previously stated:

“The rates for PTSD and cPTSD symptoms above the cut-off were quite similar with a prevalence of 4.4% (n = 88) and 4.1% (n = 83) respectively.”

The corrected sentence appears below:

“The rates for PTSD and cPTSD symptoms above the cut-off were quite similar with a prevalence of 1.4% (n = 29) and 2.5% (n = 51) respectively.”

A correction has been made to the **Discussion** section, Paragraph 1.

This sentence previously stated:

“Our results show that a third of our sample suffer from ACEs (about 24% had one to three and about 11% four or more ACEs), and about 4.1% and 4.4% fulfilled self-reported PTSD and cPTSD criteria respectively.”

The corrected sentence appears below:

“Our results show that a third of our sample suffer from ACEs (about 24% had one to three and about 11% four or more ACEs), and about 2.5% and 1.4% fulfilled self-reported PTSD and cPTSD criteria respectively.”

The authors apologize for this error and state that this does not change the scientific conclusions of the article in any way. The original article has been updated.

